# Policy-relevant differences between secondhand and thirdhand smoke: strengthening protections from involuntary exposure to tobacco smoke pollutants

**DOI:** 10.1136/tc-2023-057971

**Published:** 2023-06-01

**Authors:** Georg E Matt, Lydia Greiner, Rachael A Record, Heather Wipfli, Jamie Long, Nathan G Dodder, Eunha Hoh, Nicolas Lopez Galvez, Thomas E Novotny, Penelope J E Quintana, Hugo Destaillats, Xiaochen Tang, Antoine M Snijders, Jian-Hua Mao, Bo Hang, Suzaynn Schick, Peyton Jacob, Prue Talbot, E Melinda Mahabee-Gittens, Ashley L Merianos, Thomas F Northrup, Lara Gundel, Neal L Benowitz

**Affiliations:** 1 Department of Psychology, San Diego State University, San Diego, CA, USA; 2 School of Communication, San Diego State University, San Diego, CA, USA; 3 Keck School of Medicine, University of Southern California, Los Angeles, CA, USA; 4 Public Health Law Center, Mitchell Hamline School of Law, University of Minnesota, St Paul, MN, USA; 5 School of Public Health, San Diego State University, San Diego, CA, USA; 6 Indoor Environment Group, Lawrence Berkeley National Laboratory, Berkeley, CA, USA; 7 Biological Systems and Engineering Division, Lawrence Berkeley National Laboratory, Berkeley, CA, USA; 8 School of Medicine, University of California San Francisco, San Francisco, CA, USA; 9 Department of Molecular, Cell, and Systems Biology, University of California Riverside, Riverside, CA, USA; 10 Department of Pediatrics, Division of Emergency Medicine Cincinnati Children’s Hospital Medical Center, University of Cincinnati College of Medicine, Cincinnati, OH, USA; 11 School of Human Services, University of Cincinnati, Cincinnati, OH, USA; 12 Department of Family & Community Medicine, University of Texas Health Science Center at Houston, McGovern Medical School, Houston, TX, USA

**Keywords:** Secondhand smoke, Carcinogens, Economics, End game, Public policy

## Abstract

Starting in the 1970s, individuals, businesses and the public have increasingly benefited from policies prohibiting smoking indoors, saving thousands of lives and billions of dollars in healthcare expenditures. Smokefree policies to protect against secondhand smoke exposure, however, do not fully protect the public from the persistent and toxic chemical residues from tobacco smoke (also known as thirdhand smoke) that linger in indoor environments for years after smoking stops. Nor do these policies address the economic costs that individuals, businesses and the public bear in their attempts to remediate this toxic residue. We discuss policy-relevant differences between secondhand smoke and thirdhand smoke exposure: persistent pollutant reservoirs, pollutant transport, routes of exposure, the time gap between initial cause and effect, and remediation and disposal. We examine four policy considerations to better protect the public from involuntary exposure to tobacco smoke pollutants from all sources. We call for (a) redefining smokefree as free of tobacco smoke pollutants from secondhand *and* thirdhand smoke; (b) eliminating exemptions to comprehensive smoking bans; (c) identifying indoor environments with significant thirdhand smoke reservoirs; and (d) remediating thirdhand smoke. We use the case of California as an example of how secondhand smoke-protective laws may be strengthened to encompass thirdhand smoke protections. The health risks and economic costs of thirdhand smoke require that smokefree policies, environmental protections, real estate and rental disclosure policies, tenant protections, and consumer protection laws be strengthened to ensure that the public is fully protected from and informed about the risks of thirdhand smoke exposure.

**What is already known on this topic**

- Toxic chemical residue from tobacco smoke (thirdhand smoke, THS) can persist in indoor environments for years.

**What this study adds**

- THS contains 26 listed compounds known by the state of California to cause cancer, birth defects or other reproductive harm.
- Existing smokefree policies provide limited protection against exposure to THS.

**How this study might affect research, practice or policy**

- Smokefree policies have to be updated to protect against exposure to THS.

## Introduction

 Following decades of research linking secondhand smoke (SHS) exposure to increased morbidity and mortality risks, smokefree policies have become a leading tool to protect the public from tobacco-related disease and death.^1^ Starting in the 1970s and 1980s, individuals, businesses and the public have increasingly benefited from policies prohibiting smoking indoors, such as workplaces, public transportation, government buildings, hospitals, schools, restaurants and bars. These policies include public-sector regulations through federal, state and municipal codes, as well as voluntary private-sector rules introduced by businesses (eg, hotel groups, rental car companies), property managers and homeowners’ associations. In California alone, such smokefree policies have successfully protected the public from SHS exposure and saved thousands of lives and billions of dollars in healthcare expenditures.^1 2^ These policies, however, may not protect the public from exposure to the persistent and toxic chemical residues from tobacco smoke (also known as thirdhand smoke, THS) that can linger in indoor environments years after smoking has stopped.^3 4^ Moreover, these policies do not address the economic costs that individual consumers, businesses and taxpayers bear in their attempts to remove this toxic residue from indoor spaces and personal properties.^5^,^8^ As local, state, national and global tobacco control communities prepare for the *tobacco endgame*, policies targeting THS present an opportunity to strengthen protections against involuntary exposure to tobacco smoke pollutants from all sources, denormalise commercial tobacco use, enlist new allies and broaden the support for smokefree policies.^9^,^12^

The term *thirdhand smoke* was first mentioned in the news media in 2006 and introduced to the scientific literature in 2009.^13 14^ Funded by California’s Tobacco-Related Disease Research Program, the Collaborative Consortium on Thirdhand Smoke was created in 2011 and charged with characterising the chemical nature of THS, developing environmental indicators and biomarkers of exposure and harm, studying its health effects, exploring remediation strategies, educating the public, and devising evidence-based policies to prevent THS pollution and exposure.^15^ The Consortium has defined THS as ‘… residual tobacco smoke pollutants that remain on surfaces and in dust after tobacco has been smoked, are re-emitted into the gas phase, or react with oxidants and other compounds in the environment to yield secondary pollutants’.^3^

Research conducted by the Consortium and others has identified 26 chemicals in THS that are classified by the state of California as causes of cancer, birth defects or reproductive harm (see table 1), including some of the same carcinogens found in SHS (eg, *N*-nitrosonornicotine (NNN), nicotine-derived nitrosamine ketone (NNK)).^16^,^18^ Studies conducted in real-world field settings in the USA, Spain, China, South Korea and Germany have shown that THS can be a pervasive indoor pollutant in homes, cars, hotel rooms, casinos, movie theatres, internet cafes, university classrooms and even hospital settings that can persist for years after smoking stops in these settings.^19^,^34^

**Table 1:** Chemicals found in thirdhand smoke that are identified by California’s Safe Drinking Water and Toxic Enforcement Act of 1986 (Proposition 65) to cause cancer, birth defects or other reproductive harm, and their classification by the IARC^1869 84 91 120^,^130^

Name of chemical	CAS* number	Type of toxicity (Proposition 65)†
IARC classification 1: carcinogenic to humans		
Benzene	71-43-2	Cancer, developmental, male reproductive
Benzo(*a*)pyrene	50-32-8	Cancer
Cadmium	7440-43-9	Cancer, developmental, male reproductive
Formaldehyde	50-00-0	Cancer
NNK, 4-(methylnitrosamino)-1-(3-pyridyl)-1-butanone	64091-91-4	Cancer
NNN, *N*-nitrosonornicotine	16543-55-8	Cancer
IARC classification 2A: probably carcinogenic to humans		
Dibenz(*a,h*)anthracene	53-70-3	Cancer
Dichloromethane or methylene chloride	75-09-2	Cancer
Styrene	100-42-5	Cancer
IARC classification 2B: possibly carcinogenic to humans		
Acetaldehyde	75-07-0	Cancer
Acrylonitrile	107-13-1	Cancer
Benz[a]anthracene	56-55-3	Cancer
Benzo(*b*)fluoranthene	205-99-2	Cancer
Benzo(*k*)fluoranthene	207-08-9	Cancer
Chrysene	218-01-9	Cancer
Ethylbenzene	100-41-4	Cancer
Furan	110-00-9	Cancer
Furfuryl alcohol	98-00-0	Cancer
Indeno(*1,2,3*-cd)pyrene	193-39-5	Cancer
Isoprene	78-79-5	Cancer
Lead	7439-92-1	Cancer, developmental, female and male reproductive
*N*-nitrosopyrrolidine	930-55-2	Cancer
Naphthalene	91-20-3	Cancer
Pyridine	110-86-1	Cancer
IARC classification 3: not classified as to its carcinogenicity to humans		
Toluene	108-88-3	Developmental
California Proposition 65 classified only		
Nicotine	54-11-5	Developmental

* CAS number: Chemical Abstract Service number, a unique identifying number for chemical compounds.

† Proposition 65 requires the state of California to maintain and update a list of chemicals known to the state to cause cancer, birth defects or reproductive harm.

IARCInternational Agency for Research on Cancer (WHO)NNKnicotine-derived nitrosamine ketone

Because SHS is the precursor of THS, indoor environments where SHS is more prevalent and communities with higher smoking rates will also be at increased risk of THS exposure. THS disproportionately affects the most vulnerable populations, including children, the elderly and immunocompromised persons, as well as lower-income communities living in older, lower-quality and multiunit housing, where THS is most likely to have accumulated.^35^,^40^ A recent study showed that 95% of children <12 years old living in smokefree homes in Cincinnati, Ohio, had THS residue on their hands, and these measurements were significantly higher among children from the lowest income households.^41^ A study of 220 apartments in low-income multiunit housing in San Diego County, California, found THS residue in 100% of all non-smoker and smoker units, and 10% of non-smoker units had THS at high levels equalling those found in studies of homes of currently active smokers.^23^ A study in subsidised multiunit housing in Columbus, Ohio, showed even higher levels in both units with and without voluntary smoking restrictions compared with those in San Diego, California.^32^ Given that over 80% of the world’s 1.3 billion tobacco users live in low-income and middle-income countries, it is likely that communities in these settings are particularly affected by THS.^42^

Because THS is widespread in indoor environments, THS exposure is of concern as a significant health risk.^443^,^45^ These risks are of particular concern in children, for whom dust is a well-recognised source of exposure to environmental pollutants.^46^,^49^ Compared with adults, children spend more time indoors and interact more with THS pollution in their environment (eg, crawling on floors where dust collects, putting objects in their mouths, pica behaviour). Moreover, differences in inhalation patterns (eg, faster breathing rate relative to body weight) and immature immune systems make children more vulnerable to environmental pollutants.^50^,^53^

Laboratory studies have shown that THS exposure induces cellular DNA damage, stress responses and oncogenic phenotypes.^54^,^56^ Cytotoxicity of THS was demonstrated using mouse neural stem cells, human dermal fibroblasts, human palatal mesenchyme cells and lung A549 epithelial cells.^57 58^ Exposure to THS at very low concentrations was shown to cause distinct metabolic changes in two different types of male reproductive cell lines.^59^ In vivo studies in laboratory animals have demonstrated that THS exposure increases lung cancer risk, alters behavioural phenotypes, impairs wound healing and causes changes to the immune system.^60^,^65^

Controlled clinical human exposure studies have shown that acute inhalation exposure to THS causes changes in the human nasal epithelial transcriptome (eg, upregulated DNA repair mechanisms, stress-induced mitochondrial hyperfusion).^66^ A recent proteomics study of dermal exposure to THS in humans revealed alterations in numerous cellular pathways indicative of the potential to cause diseases (eg, atherosclerosis, inflammatory response, dermatitis), changes similar to those found in cigarette smokers.^67^ A recent study of chronic human exposure to the lung carcinogen NNK at levels found in THS and through THS-relevant exposure pathways (including dermal uptake) calculated daily doses exceeding the established *no significant risk levels*.^44^

These findings are consistent with the cancer risk assessment of exposure to tobacco-specific nitrosamines (TSNAs) in THS by Ramirez and colleagues.^31^ Based on house dust samples collected in northeastern Spain, they estimated that cancer risks through THS exposure to TSNAs at an early life stage (1–6 years) exceeded the upper-bound risk recommended by the US Environmental Protection Agency in 77% of smokers’ and 64% of non-smokers’ homes.^68^ Using the metric of disability-adjusted life years lost due to illness and death, Sleiman and colleagues^69^ compared the chronic harm caused by inhalation of specific SHS and THS constituents (ie, respirable particulate matter <2.5 µm, select volatile organic compounds (VOCs)). Under the assumption that the transition of SHS to THS was completed after 2 hours, they estimated that THS would account for 60% and SHS for 40% of the damage caused by cigarette smoke exposure.^69^ In a prospective cohort study of Chinese female never-smokers, Wen and colleagues^70^ examined cervical cancer risk of SHS and THS exposure. They concluded that the reported SHS and THS exposure was associated with a higher risk of cervical cancer. Compared with SHS and THS exposure, cervical cancer incidence was 25% higher for THS exposure and 29% higher for SHS and THS exposure. In addition, a dose–response relationship was found between exposure duration and this risk. Mahabee-Gittens and colleagues^71^ studied the clinical outcomes in children of smokers presenting to paediatric emergency or urgent care. After controlling for urinary cotinine, children with higher levels of THS exposure (as measured by nicotine level on hands) were found to be at increased risk of having discharge diagnoses previously thought to be associated only with SHS exposure, such as viral/other infectious illnesses, pulmonary illnesses and bacterial infections.

Research from controlled laboratory and real-world field studies to date has demonstrated that THS is much more than a ‘mere nuisance’.^3^ Considering that the *2006 US Surgeon General’s Report on the Health Consequences of Involuntary Exposure to Tobacco Smoke* concluded ‘there is no risk-free level of exposure’, it should not be surprising that the disease processes associated with THS exposure resemble those identified for SHS.^72^ The present evidence suggests that THS exposure is likely to contribute to human illness, but the epidemiology is still in its early stages.

Although significant progress has been made over the past 20 years, important challenges in our understanding of THS exposure, health risks and economic impacts remain. Among the key challenges are the development of specific markers of exposure to THS, distinguishing SHS and THS exposure outside of controlled laboratory settings, identifying population-level risk due to inhalation, dermal and ingestion exposures, and implementing cost-effective remediation procedures. Given the strength of the existing evidence, these challenges should not delay a careful review of existing smokefree policies in order to identify loopholes and to protect non-smokers, especially children, from involuntary exposure to THS. THS creates chronic hazardous environmental conditions where people are born, live, work, play and age that may affect a wide range of health, functional and quality of life outcomes.^73^

## Policy-relevant differences between secondhand and thirdhand smoke

The chemical constituents, physical properties and exposure routes of SHS and THS differ in fundamental ways. Because of these differences, policies that effectively protect the public from SHS may be largely ineffective against THS exposure. In the following narrative, we discuss five such differences between SHS and THS exposure to highlight gaps in current policies and how they can be closed to comprehensively protect the public from all forms of tobacco smoke exposure. While similar principles apply to the chemical residue left behind by electronic nicotine delivery devices and by cannabis consumption through smoking, vaping or dabbing, our discussion will focus on combustible tobacco products (eg, cigarettes, cigars, pipe tobacco) for which the scientific evidence is currently more advanced.^74^,^77^

### Persistent pollutant reservoirs

SHS is a complex and dynamic mixture of thousands of chemicals released into the air from the smouldering tip of a cigarette, cigar or pipe (ie, sidestream smoke) and exhaled mainstream smoke.^1 78^ Even though some SHS gases and particulate matter may be removed from the air by ventilation, the semivolatile and particulate matter constituents adsorb onto surfaces, accumulate in dust and become embedded in materials.^4 69^ If smoking continues over months and years, persistent pollutant reservoirs can develop, accumulating toxic residues deep inside materials (eg, carpet, upholstery and wallboard). Once embedded in these materials, the pollutants’ re-emission will be slowed (ie, diffusion from the core to the surface and then to the air), and they are partly protected from ambient oxidants and secondary reactions while embedded.^79^,^82^ Such reservoirs have been demonstrated in private homes after smokers adopted indoor smoking bans, moved out or quit smoking.^25 27 29^ Similarly, such reservoirs have been identified in used cars, rental cars and hotel rooms previously used by smokers.^26 28 83^ Smokers themselves become reservoirs for THS as pollutants cling to their clothes, skin and hair.^84 85^ THS pollutants continue to react with common ambient oxidants (eg, ozone, nitrous acid) and other compounds, become more toxic over time, and create novel compounds, some of which were not present in freshly emitted smoke.^5886^,^89^ While SHS pollutes the air, THS contaminates entire indoor environments, and this contamination needs to be considered as a consumer protection issue (eg, real estate, housing, used car sales, peer-to-peer commerce) and as an environmental waste management issue (eg, disposal of polluted building materials).^5 90^

### Pollutant transport

SHS pollutants are transported through the air and so are THS pollutants that are re-emitted from their reservoirs. Aerosolised particles have been shown to absorb THS contaminants and disperse them throughout a building.^91^ However, THS pollutants can also be transported through the movement of the reservoirs themselves. That is, as used cars are sold, furniture is donated, smokers come back from their breaks, old carpets are disposed of, and preowned toys, books and other personal property change hands, THS pollutants are transported in their reservoirs to new locations, including spaces that would otherwise be smokefree. A seminal study demonstrated how a smokefree movie theatre became a reservoir of THS through the transport of pollutants on the clothes and bodies of smokers.^84^ The re-emitted VOCs from THS reservoir were estimated to expose occupants to an equivalent of 1–10 cigarettes of SHS. THS transport by staff, patients or visitors is also a probable reason why THS residue is found in hospital settings, including neonatal intensive care units.^19 20 22^

### Routes of exposure

SHS exposure occurs through inhalation, while exposure to THS involves two additional exposure routes. THS pollutants can be ingested from polluted hands, objects, dust or food. Dust intake in children is a well-recognised source of exposure to environmental pollutants.^46 47^ THS pollutants can also be dermally absorbed through skin-to-skin contact with smokers (eg, parent-to-child), object-to-skin contact with polluted surfaces and materials (eg, clothes, furniture, pillows and blankets), and air-to-skin absorption from re-emitted THS. Dermal nicotine uptake directly from the air and indirectly from clothing was recently demonstrated in several studies.^44 67 92 93^

### Time gap between cause and effect

SHS exposure always occurs in relatively close temporal and spatial proximity to active smoking. This co-occurrence allows people to take action, such as moving away from the source or asking someone to stop smoking. In contrast, THS pollutants can be generated days, months and even years before potential human exposures. Furthermore, THS exposure can occur in locations (eg, a smokefree hospital and movie theatre) that are far removed from the location where the smoking took place (eg, the home or car of a visitor or employee). Imposing a smoking ban and asking someone to stop smoking are ineffective approaches to protecting against THS exposure if THS is already present or can be transported to a smokefree environment.

### Remediation and disposal

Efforts to prevent, reduce and remove SHS from the air have focused on smokefree indoor policies and increasing ventilation to replace polluted air with clean air. These approaches, however, provide only *limited* relief for THS exposure because its pollutants are stored in, and released from, indoor reservoirs that persist despite smokefree conditions. Different from SHS, remediating THS pollution requires the identification of reservoirs and cleaning or removing them to avoid exposure. Such efforts could include deep-cleaning and vacuuming of carpets; removing and disposing of polluted carpets and furniture; and fully remodelling a home or workspace, replacing floors, walls, furniture, insulation and heating, ventilation, and air conditioning (HVAC) system components. A recent field study^8^ of the effectiveness of remediation strategies has shown that standard professional cleaning approaches (eg, washing and wiping surfaces, deep-cleaning carpets, vacuuming with a high efficiency particulate air filter) provide only temporary reductions in THS, and it is re-emitted from the remaining reservoirs (eg, carpet backing, HVAC ducts, upholstery) after cleaning. This is consistent with case evidence from apartment tenants discovering excessive levels of THS 10 years after a smoker quit or passed away.^23^ The disposal of THS-polluted objects involves toxic hazardous waste (ie, carcinogens, reproductive toxicants) for which municipal landfills are not designed and which may require special disposal practices. The disposal of THS-polluted items may be one contributing factor to the identification of markers of tobacco smoke pollutants (eg, nicotine, cotinine, 3′-hydroxycotinine and *N*-formylnornicotine) in landfill leachates.^94^,^97^

## Implications for smokefree policies

Because THS is a consequence of SHS, some of our recommendations focus on strengthening and broadening existing indoor smoking bans. In addition, the differences between the properties of SHS and THS, exposure pathways and remediation efforts call for complementary approaches to protect the public from involuntary exposure to persistent THS pollutants. We highlight four policy considerations to enhance best practice strategies for protecting the public from involuntary exposure to tobacco smoke pollutants from all sources.

### Redefining smokefree as free of tobacco smoke pollutants

We call for expanding the goal of smokefree policies to achieve indoor spaces that are free of tobacco smoke pollutants in the air, on surfaces, in dust and embedded in materials. With a few exceptions, existing policies focus on smokefree air, but smokefree air policies do not guarantee freedom from THS pollutants. These policies do not address reservoirs of pollutants that accumulated during previous periods of active smoking and do not prevent the transport of THS pollutants from one space where smoking occurred into a space with a smokefree policy.^98^ This is one of the reasons why some hospital systems (eg, Cleveland Clinic, Penn Medicine)^99 100^ have ‘non-smoking hiring’ policies. Others have adopted ‘tobacco-free at work’ policies (including all breaks)^101^ and recommended handwashing and personal protective equipment to prevent THS pollution of sensitive environments (eg, neonatal intensive care units).^19 20^

### Identifying indoor environments with THS reservoirs

We call for smokefree policies to be updated to require the identification of indoor spaces polluted with THS. This can be done in two distinct ways. First, THS-polluted spaces can be identified through the disclosure of the history of smoking in the space, such as whether a previous homeowner smoked, a used car had been smoked in or multiunit housing policies previously allowed tenants to smoke inside their units. Second, THS-polluted environments can be identified through testing for the presence and level of THS. The most widely used methods require analysing surface wipe, dust or material samples for tobacco-specific markers such as nicotine, cotinine, nicotelline or TSNAs.^4^ Nicotine contamination of surfaces is the most widely used THS marker, and data are available from many different field settings that describe its distribution in smoking and non-smoking environments.^102^ Highly sensitive tests for THS markers in dust and on surfaces are available in some research laboratories, but there are currently no validated do-it-yourself tests that are available or practical for consumer use, although several are under development. Calling for mandatory disclosure and testing procedures will spur the development of sensitive and low-cost tests to allow individuals to make informed decisions about occupying, purchasing or using such spaces. In locations with high smoking prevalence or with weak indoor smoking bans and poor enforcement, testing of indoor spaces is likely to reveal exceedingly high levels of THS pollution and exposure. As such, testing data could provide compelling evidence to strengthen existing laws and enforcement, especially in low-income and middle-income countries.^103^

### Eliminating exceptions to comprehensive indoor smoking bans

We call for closing loopholes in existing smokefree policies that exempt certain indoor spaces used by the public (table 2). Such exceptions are common worldwide, especially in lower-income countries in which venues may be used for multiple purposes throughout the day. In each case, THS pollutants accumulate during periods of active smoking and then create exposure risk later when non-smokers use this space, even in the absence of smoking. Given the chemical properties of THS pollutants, the only cost-effective remedy is a comprehensive smoking ban after existing THS pollution has been removed.

**Table 2:** Examples of select indoor spaces with smoking ban exceptions that create THS exposure risks

Setting	Smoking ban	Smoking exemptions	
Home childcare, foster and group homes	When children are present.	After hours or on weekends when children are absent*.	
Home healthcare	When home healthcare provider is present.	When provider is absent.	
Hotels, online lodging market-places	Some guest rooms are smokefree.	Guests smoke in designated rooms, transporting THS through shared ventilation.	
Motor vehicles	When a minor (<18 years old) is present.	When no children are in the vehicle.	
Rental cars, party buses, limousines	Some vehicles are smokefree.	Passengers smoke in designated vehicles.	
Vehicle fleets, cabs of trucks and tractors	When non-smoking passengers are present.	Drivers smoke when alone in the vehicle.	
Workplaces	Smokefree indoor workspaces with provided smoking breaks.	Employees smoke outside when on break, transporting THS to smokefree indoor spaces on self.	
Restaurants and bars	Smokefree during the day.	Operate as hookah or smoking lounge in the evening.	

* Some jurisdictions (eg, California, New York) have already dropped this exemption.

THSthirdhand smoke

### Remediating THS pollution

We call for policies to directly address THS pollution remediation. Assessing the presence of THS is not unlike that of a home inspection after a fire to determine how much damage has occurred and which remedies are necessary to ensure a safe indoor environment. Existing research suggests that common remediation efforts (eg, carpet cleaning, repainting walls, wiping surfaces) achieve only temporary success and that persistent pollutant reservoirs need to be replaced or removed.^8^ In severe cases, THS remediation may require a complete home renovation, including the replacement of drywall, floors, insulation, built-in furniture and HVAC system components. Given substantial clean-up costs, an important part of any policy discussion must address the question of who is responsible for paying for these remediation efforts. The renters, guests and leaseholders who leave behind THS, and the housing, hotel and car fleet managers who fail to enforce compliance with smokefree policies should share some responsibility for remediation costs, an approach already applied by hotel chains and car rental companies.^104^,^106^ However, much of the pre-existing THS pollution was not caused by individuals violating smokefree policies or disregarding guidelines for the use of tobacco products. Instead, indoor smoking was promoted and smoking bans were opposed by tobacco companies that had known about toxic THS pollution caused by their products since at least the 1990s.^87^ Yet tobacco companies and retailers failed to advise consumers to refrain from indoor smoking, and their responsibility for the costs of environmental contamination caused by the use of their products should be given careful consideration.

## California policy examples

While each jurisdiction has somewhat different approaches to designing and implementing smokefree policies, it is worth considering California as an example of how various jurisdictions might update their smokefree laws to encompass THS protections, particularly in the context of real estate transactions. This includes policies surrounding (a) disclosures and professional standards and (b) remediation requirements.

### Disclosures and professional standards

As previously noted, requiring THS disclosure to new homebuyers or renters would be one approach to help inform and protect the public from existing THS residue. There are several ways in which residential THS disclosure could be implemented.

One option would be to specifically add the presence of THS as a required disclosure under state law for any residential property sale. In California, a real estate agent owes a general duty to disclose to potential buyers all facts material to a sale. This means that the agent should identify and disclose facts that are likely to affect the judgement of the buyer. Since this is open to significant interpretation, California law also requires certain categories of disclosure, in essence deeming them ‘material’. The seller of a residential property documents this information in the required *Transfer Disclosure Statement*, which is in a form specified by statute.^107^ California law could be amended to require disclosure of THS presence in the Transfer Disclosure Statement or as a separate statutorily required disclosure. This would elevate the visibility of THS as a potential issue a buyer may choose to address before closing on the purchase of a property.

A second option would be to specifically add THS to the statutory list of common environmental hazards that need to be included in the *Residential Environmental Hazards Booklet*, produced by the California Environmental Protection Agency (CalEPA). California law established this booklet, which if provided to a residential buyer is deemed adequate to have informed the buyer about ‘common environmental hazards’.^108^ While it is not mandatory to provide the booklet, it does provide certain legal protections to the seller. Providing the booklet does not, however, relieve the sellers of their obligation to disclose any known environmental hazards to a potential buyer.^109^ The booklet must discuss the significance of these hazards and what can be done to mitigate them, and it must provide sources to gather more information.^110^ Specifically, state law requires that the booklet include a description of common environmental hazards, including but not limited to ‘asbestos, radon gas, lead-based paint, formaldehyde, fuel and chemical storage tanks, and water and soil contamination’.^111^ Although the current booklet does not include a specific discussion of the hazards of THS, it does mention that tobacco smoke is a potential source of carbon monoxide in the home and that ‘combustion sources such as cigarettes’ are a potential source of formaldehyde.^112^

THS could be added through a legal amendment by passing a law requiring the inclusion of THS within the booklet or by requiring CalEPA to voluntarily include an explicit discussion of THS in the booklet. CalEPA has the discretion to include a discussion of anything it deems a ‘common environmental hazard’ under existing statutory requirements.^111^

A third option would be to pass legislation to develop a specific THS hazard booklet and then require that it be provided to all prospective buyers and renters. This is similar to the approach used in California for mould.^113^ The creation of an educational booklet dedicated entirely to THS would send a strong message about the known risks and demonstrate a commitment to increasing awareness of THS pollution in indoor environments.

### Remediation requirements

A more robust approach would be to specify that THS is a housing code violation requiring remediation. California law specifies certain conditions that, if they exist ‘to an extent that endangers the life, limb, health, property, safety, or welfare of the public or the occupants’, are considered substandard for occupation by a tenant and require remediation.^114^ This includes conditions such as the dampness of habitable rooms and visible mould growth.^115^ The owner of a substandard building can be ordered by enforcing agencies to make repairs and, if they do not make such repairs after receiving notice, face the possibility that the state steps in to remediate the conditions.^116^ Property owners can then be liable for the costs of abatement.^117^

One strategy to support this approach would be to amend the law that specifies which conditions create substandard housing to include THS. There could also be additional criteria established in law for when a property would be substandard with THS present, such as the requirement for a visible growth of mould in order to be deemed substandard, not simply any presence of mould. Requiring THS remediation would provide greater enforcement potential and a strong incentive for landlords to move towards smokefree buildings.

## Conclusions

SHS is the precursor of THS, and all policies aimed at eliminating SHS will help prevent the accumulation of new THS. Current smokefree policies, however, do not address existing THS pollution that accumulated earlier or the transport of THS pollution into smokefree environments. Two decades of THS research support widening and strengthening indoor smoking bans to create indoor environments free of tobacco smoke pollutants from SHS and THS.

As the tobacco control community works to close existing regulatory loopholes and considers *tobacco endgame* strategies, it is an opportune time to review, strengthen and expand policies designed to protect the public from exposure to existing THS that has accumulated over decades of permissive indoor smoking policies. Recognising THS as a novel and distinct health risk can contribute to closing loopholes and promoting *tobacco endgame* strategies in three major ways. First, THS provides novel arguments as to why comprehensive smoking bans in all indoor environments are warranted, should not be delayed and may not have exemptions. Indoor smoking bans not only protect current occupants from SHS exposure but also future occupants from THS exposure and the property owner from remediation costs. This is especially relevant in low-income and middle-income countries where partial smoking bans remain common.^103^ Second, the many implications of THS demonstrate the broad societal impact of commercial tobacco use and bring new allies to tobacco control efforts that traditionally have not been part of the tobacco control movement. This includes renters and homebuyers, realtors and property managers, used car sellers and buyers, home inspectors, insurance companies, and lenders. New allies also include environmental organisations concerned about the disposal of THS-polluted building materials, carpets and furniture and environmental justice organisations concerned about the social determinants of health associated with where people live, work and play. Third, THS will linger in indoor environments long after the last cigarette has been smoked. Current tobacco control strategies should address how to deal with such indoor environments, including how they can be identified, how they can be remediated and who should pay for the costs.

The toxic legacy of tobacco smoke contributes to health inequities by disproportionately affecting the most vulnerable populations, including children, the elderly and immunocompromised persons.^23 41^ Further, lower-income and marginalised communities are most likely to reside in older, lower-quality and multiunit housing, where THS is most likely to have accumulated, posing increased health risks.^118 119^ Smokefree policies, environmental protections, real estate and rental disclosure policies, tenant protections, and consumer protection laws should be reviewed and updated to ensure that the public is fully protected from and informed about the risks of THS exposure. Proponents of environmental sustainability and environmental justice can become valuable allies in the effort to prevent exposure to toxic tobacco residues that leach into the soil and water supplies when polluted goods, including building materials, are disposed of in landfills. THS is a form of tobacco product waste, and the manufacturers, suppliers and retailers of commercial tobacco products should assume responsibility to prevent and mitigate the persistent toxic legacy of their products.
